# Development of Perphenazine-Loaded Solid Lipid Nanoparticles: Statistical Optimization and Cytotoxicity Studies

**DOI:** 10.1155/2021/6619195

**Published:** 2021-04-28

**Authors:** Parisa Abbasi Farsani, Reza Mahjub, Mojdeh Mohammadi, Seyed Sajad Oliaei, Mohammad Mehdi Mahboobian

**Affiliations:** ^1^Department of Pharmaceutics, School of Pharmacy, Hamadan University of Medical Sciences, Hamadan, Iran; ^2^Department of Pharmacology & Toxicology, School of Pharmacy, Hamadan University of Medical Sciences, Hamadan, Iran; ^3^Department of Medicinal Chemistry, School of Pharmacy, Medicinal Plants & Natural Products Research Center, Hamadan University of Medical Sciences, Hamadan, Iran

## Abstract

**Objective:**

Perphenazine (PPZ), as a typical antipsychotic medical substance, has the same effectiveness compared to atypical antipsychotic medications for the treatment of schizophrenia. Despite the lipophilic essence, PPZ encounters limited bioavailability caused by the first-pass metabolism following oral administration. In the present study, PPZ-containing solid lipid nanoparticles (PPZ-SLNs) were prepared and optimized based on different factors, including lipid and surfactant amount, to develop appropriate and safe novel oral dosage forms of PPZ.

**Methods:**

The solvent emulsification-evaporation method was utilized to form SLNs by using soybean lecithin, glycerol monostearate (GMS), and Tween 80. Statistical optimization was done by the Box-Behnken design method to achieve formulation with optimized particle size, entrapment efficiency, and zeta potential. Also, transmission electron microscopy, *in vitro* release behavior, differential scanning calorimetry (DSC), and powder X-ray diffractometry (P-XRD) studies and cytotoxicity studies were assessed.

**Results:**

Optimization exhibited the significant effect of various excipients on SLN characteristics. Our finding indicated that the mean particle size, zeta potential, and entrapment efficiency of optimized PPZ-SLN were, respectively, 104 ± 3.92 nm, −28 ± 2.28 mV, and 83% ± 1.29. Drug release of PPZ-SLN was observed to be greater than 90% for 48 h that emphasized a sustained-release pattern. The DSC and P-XRD studies revealed the amorphous state of PPZ-SLN. FTIR spectra showed no incompatibility between the drug and the lipid. Performing cytotoxicity studies indicated no significant cytotoxicity on HT-29 cell culture.

**Conclusion:**

Our study suggests that PPZ-SLNs can make a promising vehicle for a suitable therapy of schizophrenia for the oral drug delivery system.

## 1. Introduction

Schizophrenia is a debilitating disorder with heterogeneous symptoms that can cause various social, mental, and functional impairments [1]. For the last two decades, the disease has been categorized as one of the leading causes of disability in human life [2]. Relevant reports from the World Health Organization indicated that approximately 21 million people worldwide suffered from the disease in the year 2016, and according to epidemiological studies, the prevalence of the disorder is higher in developing countries rather than developed ones [3].

Due to the essential role of dopamine in the development of schizophrenia, blocking D2/D3 receptors by antipsychotic compounds is considered an effective therapeutic strategy [4]. Currently, the first and the second generations of dopamine antagonists, respectively, designated as typical and atypical antipsychotic drugs, are prescribed as substantial therapy [5]. Perphenazine (PPZ), as a typical antipsychotic, is a lipophilic phenothiazine derivative compound, with a partition coefficient of 4.2 and aqueous solubility of less than 0.1 mg/ml which is mainly metabolized through hepatic pathways. These characteristics result in low bioavailability through oral administration [6–9]. Although different approaches have been proposed to improve the low oral bioavailability of PPZ, including the development of sublingual solid dispersion [10], fast disintegrating tablets [11], electrospun fibers [12], and ultrafine oil-water emulsions [13], recent developments in nanotechnology offer abundant possibilities for the preparation of more efficient oral drug delivery systems to overcome these problems [14]. Among various nanostructures, solid lipid nanoparticles (SLNs) as an alternative drug carrier represent considerable advantages, including enhanced bioavailability, elaborated stability and drug payload, facilitated drug delivery to the brain, and a high potential for commercial manufacturing as well as the high possibility for drug targeting and lack of biotoxicity [15–20].

SLN nanospheres are composed of a physiologically safe surfactant such as Tweens, bile salts, and phospholipids mixed with a lipid matrix with an intrinsic tendency to solidify at room temperature (e.g., waxes, fatty acids, and glycerides). Particles in the size range of 120-200 nm are most preferred for efficient drug delivery to the central nervous system (CNS) to reduce the prompt clearance of the nanospheres in the bloodstream through the reticuloendothelial system (RES) [21, 22]. Facilitating the drug transport through the lymphatic pathway and therefore bypassing the portal circulation along with high stability in a gastrointestinal tract make PPZ-containing SLNs an alternative strategy for reduction of the first-pass metabolism and increase the oral bioavailability of the drug [23–25]. Moreover, due to their lipophilic features and their appropriate size, SLNs can be considered a desirable colloidal drug carrier that can easily penetrate CNS [26, 27]. Therefore, the formulation of capsules and compressed tablets containing drug-encapsulated SLNs can be accounted for as a successful effort to diminish erratic absorption, improve bioavailability, and increase lymphatic uptake [28, 29].

The objective of this study was to develop PPZ-containing solid lipid nanoparticles (PPZ-SLNs) using the emulsion-solvent evaporation technique. To achieve optimized nanoparticles, an experimental design based on response surface methodologies was applied, and the physicochemical properties of nanoparticles, including size, zeta potential, and encapsulation efficiency, were examined. Furthermore, morphology, *in vitro* release studies, and solid phase characterizations were performed. Finally, cytotoxicity of optimized nanoparticles on the HT-29 cell line, as a valuable representative for intestinal cells such as enterocytes and mucus-producing cells, was investigated [30].

## 2. Materials and Methods

### 2.1. Chemicals

Perphenazine (PPZ) was obtained from Darou Pakhsh Pharmaceutical Co. (Tehran, Iran). Tween 80 and lecithin were provided from Samchun Pure Chemical Co., Ltd. (South Korea). Glycerol monostearate (GMS) was gifted from Gattefosse Co. (Lyon, France). Chloroform and dichloromethane were obtained from Duksan Pure Chemicals (South Korea). Ammonium acetate was purchased from Carlo Erba Reagents (France). Analytical grade methanol and acetonitrile were obtained from Merck (Darmstadt, Germany). For cell culture studies, penicillin-streptomycin (1%), Phosphate-Buffered Saline (PBS), dimethyl sulfoxide (DMSO), Roswell Park Memorial Institute (RPMI), methylthiazolyldiphenyl-tetrazolium bromide (MTT), Fetal Bovine Serum (FBS), and trypsin were all obtained from Sigma-Aldrich (Wisconsin, United States). Deionized water was freshly obtained by a Millipore® Ultrapure Water purification system.

### 2.2. Preparation of PPZ-SLNs

PPZ-SLNs were prepared by the solvent emulsification and evaporation technique. Briefly, the aqueous phase consisted of various amounts of Tween 80 which was previously dissolved in 25 ml of purified water and was heated to 70°C using a Memmert® water bath (Germany). On the other hand, the nonaqueous phase (organic phase) consisted of PPZ (5 mg) as the therapeutic ingredient, which was mixed with different amounts of GMS and lecithin and was dissolved in combination with chloroform (1.5 ml) and dichloromethane (0.5 ml) under 5-minute sonication to obtain a transparent organic phase. For complete dissolution, the mixture was heated to 70°C in a water bath. The prepared organic phase was added dropwise into the aqueous phase under the homogenization at 10,000 rpm at 70°C using a Heidolph® homogenizer (Germany). The resultant opaque emulsion was kept agitated under the same homogenizer speed for one hour. Subsequently, SLNs were obtained by cooling samples in an ice water bath for 30 minutes for quick crystallization of the lipids.

### 2.3. Characterization of PPZ-SLNs

#### 2.3.1. Determination of Particle Size and Zeta Potential

Following the preparation of PPZ-loaded SLNs, the size and polydispersity index (PDI) of freshly prepared nanoparticles were determined by photon correlation spectroscopy by Zetasizer 300HS (Malvern Instruments™, UK). Moreover, their appropriate zeta potentials were measured using laser Doppler anemometry by the same instrument. Each sample was diluted at a 1 : 10 ratio using freshly prepared double distilled water. All measurements were done in triplicate, and the instrument was operated under a constant temperature of 25°C.

#### 2.3.2. Determination of Entrapment Efficiency (EE%)

The entrapment efficiency (EE%) of PPZ in the nanoparticles was determined indirectly by the ultracentrifugation technique [31]. Briefly, a freshly prepared colloidal nanosuspension was centrifuged at 20,000 rpm for 30 min using a Beckman Coulter® ultracentrifuge (Georgia, United States). During the centrifugation, the temperature was kept constant at 4°C. Then, the settled-down nanoparticles were collected, and the transparent supernatant was analyzed for the determination of the amount of the unentrapped drug using the HPLC technique (*n* = 3). Twenty microliters of the samples was injected into a Shimadzu® high-performance liquid chromatographic system equipped with an LC-20AD pump and an SPD-20A UV detector that was previously set at 256 nm. A Hector-M® ODS column (250∗4.6∗5) as the stationary phase and a mobile phase that consisted of acetonitrile : ammonium acetate (0.01 M) : methanol (45 : 45 : 10) with a constant flow rate of 1.5 ml/min were used for liquid chromatography (Bogdanovska et al. 2014; Mandal and Ace 1993). According to ICH guidelines, partial method validation was performed in terms of specificity, linearity, accuracy, and repeatability [32].

For the quantification of PPZ, the standard curve was depicted, and the results showed the linearity over the concentration range of 0.78-25 *μ*g/ml with a correlation coefficient of 0.9988. Finally, the entrapment efficiency (EE%) of the prepared nanoparticles was calculated using the following equation:

(1) $$\begin{array}{c} \text{EE}\%=\left(1-\frac{\text{concentration of free drug in the supernatant}}{\text{concentration of total initial drug}}\right). \end{array}$$

### 2.4. Design of Experiment and Optimization

In this study, the Box-Behnken design (BBD) was utilized for the determination of the significance of linear coefficients of three independent variables (i.e., factors), including the concentration of Tween 80 (*X*_1_), the amount of lecithin (*X*_2_), and the amount of GMS (*X*_3_), as well as their squared coefficients and their binary interaction coefficients on dependent variables (i.e., responses) including particle size (*Y*_1_), zeta potential (*Y*_2_), and entrapment efficiency (*Y*_3_) of prepared SLNs. The ranges of factors and constraints which were previously determined using a set of preliminary studies are summarized in Table 1. Then, the obtained data were fitted to appropriate mathematical models, and finally, the optimized factors were predicted using Design-Expert® software (V. 8.8.1. Stat-Ease, Inc., Minneapolis, United States). According to the software and design of experiment (DoE) principles, experimental preparation of a set of 15 various formulations was sufficient to develop appropriate models, explained by second-order polynomial functions as follows:

(2) $$\begin{array}{c} Y_{A,B,C}=\beta_{0}+\beta_{1}X_{1}+\beta_{2}X_{2}+\beta_{3}X_{3}+\beta_{11}{X_{1}}^{2}+\beta_{22}{X_{2}}^{2}+\beta_{33}{X_{3}}^{2}+\beta_{12}X_{1}X_{2}+\beta_{13}X_{1}X_{3}+\beta_{23}X_{2}X_{3}, \end{array}$$

where *Y*_*A*,*B*,*C*_ are the predicted responses for particle size, zeta potential, and EE%, as physicochemical properties of nanoparticles, respectively; *β*_0_ is the intercept; *β*_1_, *β*_2_, and *β*_3_ are the linear coefficients for concentrations of Tween 80, lecithin, and GMS, respectively; *β*_11_, *β*_22_, and *β*_33_ are the squared coefficients for concentrations of Tween 80, lecithin, and GMS, respectively; *β*_12_ is the binary interaction coefficient between concentrations of Tween 80 and lecithin; *β*_13_ is the binary interaction coefficient between concentrations of Tween 80 and GMS; *β*_23_ is the binary interaction coefficient between concentrations of lecithin and GMS; *X*_1_, *X*_2_, and *X*_3_ are independent variables.

### 2.5. Freeze Drying

To prepare lyophilized nanoparticles, the optimized formulation was prepared and ultracentrifuged. The resultant sediment was suspended in deionized water (7 ml) containing 1% mannitol as cryoprotectant and stored overnight at -66°C (Operon Ultra-Low Temperature Freezer). Then, the sample was transferred to a freeze dryer (Operon®, South Korea) and was dried for 48 h under a working pressure of 0.07 bar at -55°C to achieve the fine freeze-dried sample.

### 2.6. Morphological Studies

The morphological analysis of optimized formulation was conducted using transmission electron microscopy (TEM; EM10C, Zeiss, Jena, Germany) at 100 kV. Few drops of the sample were placed on a 300-mesh carbon-coated copper grid and dried at room temperature.

### 2.7. *In Vitro* Release Studies

The dialysis bag diffusion technique and a USP paddle diffusion apparatus were applied to the study of the *in vitro* release profile of the drug from PPZ-SLNs [33]. The appropriate amount of lyophilized nanoparticles equivalent to 500 *μ*g of PPZ was dispersed in previously prepared simulated intestinal fluid (pH = 6.8) as the release medium and was transferred to a dialysis bag with a molecular cutoff of 12400 Da. The dialysis bag was immersed in 250 ml of the preheated release medium and stirred at 50 rpm. Throughout the experiment, the temperature was kept constant at 37 ± 1°C using a temperature-controlled water bath. Samples (3 ml) were taken at predetermined time intervals of up to 48 hours, and the amount of PPZ released from nanoparticles was determined using HPLC. PPZ dispersed in purified water was used as the control.

### 2.8. Solid State Characterization

#### 2.8.1. Differential Scanning Calorimetry (DSC)

Differential scanning calorimetry analysis of PPZ-SLN, bulk PPZ, pure lipid, and physical mixture (PM) of the drug and lipid (1 : 1) was performed using DSC Q600 (TA Instruments, New Castle, DE). 6 mg of each sample was weighed and transferred into a standard aluminum pan and carefully sealed; an empty pan was used as a reference. Both pans were subjected to heat over a temperature range of 40 to 200°C with a heating rate of 10°C per min. Nitrogen purge gas was used to maintain an inert atmosphere in sample cells.

#### 2.8.2. Powder X-Ray Diffractometry (P-XRD)

An X-ray diffraction study was employed to determine the crystalline states of samples. Samples used for P-XRD analysis were the pure drug (PPZ), lipid (GMS), physical mixture of the drug with lipid (1 : 1), and lyophilized PPZ-SLN. The study was carried out using an X-ray diffractometer (Malvern PANalytical BV, Netherlands). X-ray diffraction studies of samples were conducted by subjecting them to nickel-filtered CuK*α* radiation (40 kV, 40 mA), and anode material was Cu and scanned from 2° to 70°, 2*θ* at a step size of 0.026°. The study was performed at 25°C.

#### 2.8.3. Fourier Transform Infrared Spectroscopy (FTIR)

FTIR analysis was carried out using an FTIR spectrometer (Bruker, Tensor 27, Germany). 2 mg of each sample was transferred into an agate mortar; then, 200 mg of potassium bromide (KBr) was added; and the mixture was ground to obtain a fine homogenous powder. Each triturated sample of the pure drug (PPZ), lipid (GMS), physical mixture of the drug with lipid (1 : 1), and lyophilized PPZ-SLN was made into a thin and transparent pellet by applying pressure. Each pellet was scanned through a wave number range of 400-4000 cm^–1^.

### 2.9. Cytotoxicity Studies

The cytotoxicity of the optimized PPZ-loaded SLN formulations on the HT-29 cell line was assessed using MTT (3-(4,5-dimethylthiazol-2-yl)-2,5-diphenyl-2H-tetrazolium bromide) [34].

For this study, HT-29 cells with passage numbers 4 to 6 were cultured in the RPMI medium supplemented with 10% FBS and 1% penicillin-streptomycin and then incubated at 37°C in a humidified environment under CO_2_ (5%). The medium was changed every day, and the cells were subcultured after reaching 80–90% confluency.

After reaching adequate confluency, the cells were seeded in a 96-well microplate at a density of 6 × 10^3^ cells/well and incubated for 24 h to ascertain cell attachment. Various concentrations of SLNs (i.e., 0.1 *μ*M, 0.2 *μ*M, and 0.3 *μ*M) were prepared by reconstitution of the nanoparticles by RPMI, and cells were treated with 150 *μ*l of the nanosuspensions. MTT assay was performed after 24 h and 48 h post-incubation. For performing the assay, 10 *μ*l of the MTT reagent (5 mg/ml) was added to each well and incubated for three hours. Then, for solubilizing the formazan crystals, 100 *μ*l of DMSO was added to each well, and the plate was shaken by a plate shaker. After 15 min of shaking, the concentration of solubilized formazan was determined using an ELISA reader at a wavelength of 570, and the cell viability was calculated according to the following equation:

(3) $$\begin{array}{c} \%\text{cell viability}=\frac{\text{Absorbance of treatment}-\text{Absorbance of blank}}{\text{Absorbance of control}-\text{Absorbance of blank}}\times 100. \end{array}$$

### 2.10. Statistical Analysis

In this study, except for the experiments done for model validation performed five times, all other experiments were done in triplicate, and the relevant results were reported as mean ± SD. For performing the Box-Behnken experimental design, Design-Expert® software (V. 8.8.1. Stat-Ease, Inc., Minneapolis, United States) was used for modeling, prediction, and optimization. For comparison between two groups, the two-sample independent *t*-test was performed using SPSS. The level of significance was considered to be 0.05 in all statistical analyses.

## 3. Results and Discussion

### 3.1. Preparation and Characterization of Nanoparticles

Design-Expert®software was utilized to evaluate the effects of independent variables (factors), including the concentration of Tween 80 (mg/ml), amounts of GMS (mg), and amounts of lecithin (mg), on the dependent variables (responses), including size, zeta potential, and EE% of nanoparticles using Box-Behnken response surface methodology.

After experimental preparation of suggested formulations and determining the appropriate responses, the data were fitted to the best-correspondent models with a convenient correlation coefficient using the stepwise method. Statistical significance of the fitted models was affirmed using one-way analysis of variance (ANOVA), while *p* < 0.05 was considered the level of significance.

### 3.2. Characterization of PPZ-SLNs

Size, zeta potential, and entrapment efficiency are considered the most important physicochemical characteristics of colloidal systems intended for oral delivery of the drugs. In these formulations, by reducing the particle size to less than 500 nm, cellular uptake in the lymphoid system of the intestine can be improved [35, 36]. Also, the delivery system can efficiently bypass the liver first-pass metabolism [37].

#### 3.2.1. Particle Size

As shown in Table 2, nanoparticle sizes were within the range of 96.15 ± 3.37 nm (i.e., formulation no. 2) to 264.36 ± 12.37 nm (formulation no. 13). For the prediction of particle size, statistical analysis was performed by Design-Expert® to fit the data to the proper significant model. The characteristics of the fitted model are summarized in Table 3. The regression analysis of variance showed that the linear coefficients of two independent variables *A* and *C*, designated as the concentration of Tween 80 and the amount of GMS, respectively, were significant (*p* < 0.05), while the amount of lecithin (*B*) showed the nonsignificant effect on the sizes of particles. Moreover, the binary interaction between the two factors *A* and *C* showed a significant effect on nanoparticle sizes. The coefficients of significant variables on particle size (*Y*_1_) are shown as follows:

(4) $$\begin{array}{c} Y_{1}=+162.14+\left(31.11*A\right)+\left(14.09*C\right)+\left(42.72*A\cdot C\right), \end{array}$$

where *Y*_1_ is the particle size of SLNs; *A* is the linear coefficient for the concentration of Tween 80; *C* is the linear coefficient for the amount of GMS; *A* · *C* is the coefficient for binary interaction between the concentration of Tween 80 and amount of GMS.

**Figure 1:**
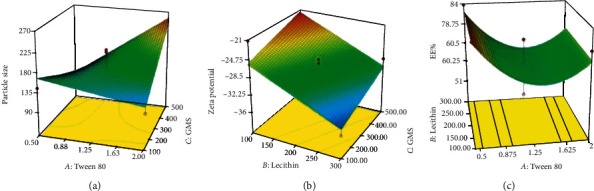
3D response surface plot showing the effect of (a) Tween 80 and GMS on size, (b) lecithin and GMS on zeta potential, and (c) Tween 80 and lecithin on EE%.

The 3D response surface plot of changes in nanoparticle sizes due to alteration in significant variables is shown in Figure 1. As illustrated, in the highest value for the concentration of Tween 80 (i.e., 2 mg/ml), by increasing the amount of GMS, the size of SLNs was increased dramatically and reached values more than 250 nm in the highest amount of GMS (i.e., 500 mg). On the other hand, in the lowest value for the concentration of Tween 80 (i.e., 0.5 mg/ml), it was observed that the size of nanoparticles declined as a result of increasing the concentration of GMS. Moreover, in the lowest value for the amount of GMS (i.e., 100 mg), the size of nanoparticles was slightly increased by raising the concentration of Tween 80; in the highest value for the amount of GMS (i.e., 500 mg), it dramatically fell as the result of increasing the concentration of Tween 80.

Ebrahimi et al. [38] investigated the effects of various surfactants on the particle size of SLNs. They revealed that using nonionic hydrophilic surfactants such as Tween 80 leads to smaller particle sizes than nanoparticles prepared by other counterparts. Similar results were obtained by Sznitowska et al. [39] and Soddu et al. [40]. In the present study, by using Tween 80 and lecithin as surfactants, nanoparticles with less than 300 nm were obtained.

The effect of blending surfactants in lowering interfacial tension causes achieving these nonmetric particle sizes [38, 41, 42]. As shown in Figure 1, by increasing the concentration of Tween 80, particle size would be reduced in the lowest amount of GMS. This phenomenon is due to a further reduction in interfacial tension and enhanced stabilization of particles by reduction of aggregation tendency as the result of higher concentrations of the surfactant [43]. In contrast, it was observed that with increasing the amount of GMS, the effect was reversed, and therefore, in the highest amount of GMS, the smallest particle size was achieved in the lowest concentration of Tween 80 (i.e., 0.5%). Moreover, it was shown that in the highest concentration of the surfactant, the particle size was dramatically increased following an increase in the amount of GMS. It is suggested that in higher amounts of GMS, the lipid tends to merge, and by increasing the viscosity of the prepared emulsion, an increase in the size of SLNs could be justified [44]. Furthermore, the increase in the concentration of the lipid phase could be attributed to a decrease in emulsifying efficiency of the surfactant and elevated particle aggregation [45].

#### 3.2.2. Zeta Potential

In this study, the zeta potential (ZP) of nanoparticles was varied between −35.8 ± 1.32 mV (formulation no. 14) and −21.2 ± 2.21 mV (formulation no. 1) (Table 2). The regression analysis of variance implies that the linear coefficients of two independent variables, *B* and *C*, designated as the amount of lecithin and the amount of GMS, respectively, posed significant effects on the ZP of the nanoparticles (*p* < 0.05). Statistical analysis was performed by Design-Expert®software, and the experimental data were fitted to the most appropriate significant model (*p* < 0.05) using the stepwise method. The characteristics of the proposed model are summarized in Table 3, and the coefficients of significant variables on ZP (*Y*_2_) are shown as follows:

(5) $$\begin{array}{c} Y_{2}=-28.009-\left(4.28*B\right)+\left(2.17*C\right), \end{array}$$

where *Y*_2_ is the zeta potential; *B* is the linear coefficient for the amount of lecithin; *C* is the linear coefficient for the amount of GMS.

The 3D response surface plot, illustrated in Figure 1, indicates that the ZP of nanoparticles was dramatically reduced as a result of increasing the amount of lecithin. Moreover, in both the highest and lowest values for amounts of lecithin, the ZP was observed to increase by increasing the GMS amounts.

ZP could be one of the most suitable indicators to ensure nanoparticle stability during storage. High values of ZP, usually more than ±30 mV, inhibit agglomeration of particles by electrical repulsion and therefore can guarantee nanoparticle stability [46, 47]. Based on the obtained results (Table 2), the ZP of all samples is between -21 and -35 mV. The presence of GMS and the zwitterionic structure of lecithin impart negative charges to the surface of nanoparticles [48, 49]. As shown in the three-dimensional graph (Figure 1), by increasing the concentration of lecithin, the ZP was increased gradually, indicating the dominant role of lecithin in negative charge density on the nanoparticle surface. Although all the formulation did not exhibit sufficient surface charges for stabilization of the particles, due to the steric stabilizing effect, the incorporation of Tween 80 can avoid particle aggregation [50]. Therefore, in this study, it is suggested that both the steric and electrostatic repulsions are responsible for particle stabilization.

#### 3.2.3. Entrapment Efficiency (EE%)

The entrapment efficiency (EE%) of the prepared SLNs was calculated and summarized in Table 2. The related data were in the range of 36.1 ± 3.76% (formulation no. 6) to 87.5 ± 1.65% (formulation no. 2). The regression analysis of variance implies that linear coefficients of factor *A* (i.e., the amount of Tween 80), as well as the squared coefficient (*A*^2^), were significant (*p* < 0.05), while other independent variables posed nonsignificant effects on EE%. Statistical analysis was performed by Design-Expert® software, and the experimental data were fitted to the most appropriate significant model (*p* < 0.05) by the stepwise method. The characteristics of the proposed model are summarized in Table 3, and the coefficients of significant variables on EE (*Y*_3_) are shown as follows:

(6) $$\begin{array}{c} Y_{3}=+65.72-\left(5.03\times A\right)+\left(13.38\times A^{2}\right), \end{array}$$

where *Y*_3_ is the EE%; *A* is the linear coefficient for the concentration of Tween 80; *A*^2^ is the squared coefficient for the concentration of Tween 80.

As shown in the 3D response surface plot (Figure 1), the EE% fell dramatically by increasing the concentration of Tween 80 up to 1.25 mg/ml. In contrast, by a further increase in the concentration of Tween 80, a significant rise in the values of EE% could be observed.

The EE% of PPZ in the nanoparticles was evaluated to determine the amount of the encapsulated drug. In this study, the achievement of high values for EE% indicated the compatibility between Tween 80 and PPZ in the lipophilic core of the SLNs. The achieved results showed that the EE% was decreased by increasing the concentration of Tween 80 up to 1.25%, suggesting that the incorporation of excess amount of the surfactant into SLNs led to a decrease in EE%. In contrast, it was observed that the EE% was increased by a further increase in the concentration of Tween 80 from 1.25 to 2.0%. The phenomenon could be justified by considering the effect of the surfactant in high concentrations on enhanced solubilization of the drug in the core of SLNs [51]. In this study, in accordance with the study performed by Kheradmandnia et al. [41], it was shown that the amount of lecithin exhibited a nonsignificant role in the EE%.

### 3.3. Optimization and Model Validation

The optimization of the physicochemical characteristics of SLNs was carried out according to statistical analysis of experimentally obtained data utilizing Box-Behnken response surface methodology. The optimized and predicted parameters for the preparation of SLNs are shown in Table 4. To determine the model validation and calculation of the appropriate prediction errors, the suggested optimized formulation was prepared and characterized experimentally (*n* = 5). The observed responses and the values of predicated errors are indicated in Table 4. As shown in the table, the calculated prediction errors were below 10% for all responses demonstrating the proper predictability, efficiency, and adequacy of the proposed models.

### 3.4. Morphological Studies

**Figure 2:**
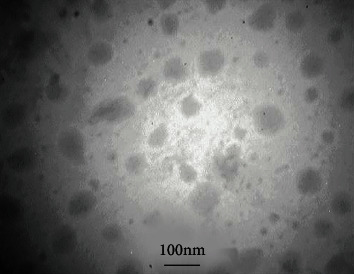
TEM image of optimized SLN.

Images obtained from transmission electron microscopy (TEM) of the optimized SLN preparation are illustrated in Figure 2. TEM images revealed the formation of nonaggregated and spherical-shaped SLNs with appropriate diameters in accordance with data obtained by photon correlation spectroscopy (PCS).

### 3.5. *In Vitro* Release Study

**Figure 3:**
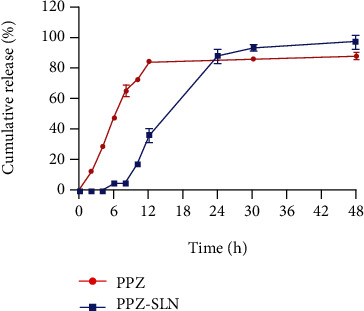
*In vitro* release profile of the optimized PPZ-loaded SLNs and pure PPZ (data shown as mean ± SD, *n* = 3).

The *in vitro* release profile of PPZ from the optimized SLNs was obtained using a dialysis membrane immersed in the simulated intestinal fluid (pH = 6.8). The data revealed a sustained and prolonged release profile with an initial lag time of approximately 6 hours and no burst release. The observed prolonged release can be justified by considering drug incorporation in the lipophilic matrix of the particles. The nanoparticles exhibited approximately complete drug release within 48-hour postincubation time (Figure 3). For determining release mechanism, the data were mathematically fitted to several release kinetic models, including zero-order, first-order, second-order, diffusion, Hixon-Crowell, and baker-Lonsdal. According to data provided in Table 5, the release mechanism was best fitted to the Hixson-Crowell model.

The rate of diffusion and the appropriate rate of dissolution are considered the two major rate-limiting mechanisms in the release of encapsulated therapeutics from SLNs [52–54]. To investigate the *in vitro* release profile, the optimized formulations were dispersed in simulated intestinal fluid, and finally, a prolonged, sustained-release profile was obtained. As mentioned earlier, PPZ has a lipophilic nature; therefore, entrapment of the compound into a lipid matrix containing GMS and lecithin could retard the drug release due to arduous diffusion. The results were in accordance with previous studies reporting the sustained-release profile of lipophilic compounds from SLNs [55–57]. In contrast to Li et al. [58], which reported the burst release of quercetin from SLNs due to surface accumulation of the compound, in this study, no burst release was observed, indicating homogenous encapsulation of PPZ in the lipid matrix of the particles. Reducing lipid crystallinity can result in more vital interaction between the drug and the lipid matrix, and therefore, the selection of an appropriate mixture of lipids can prevent the unfavorable burst release effect. Moreover, prevention in the formation of a drug-enriched shell by using lower amounts of the surfactant may be considered another reason for the observed slow release of PPZ from the nanoparticles [59]. In this study, it was found that the release profile was best fitted to the Hixson-Crowell kinetic model. Therefore, dissolution, which was determined by the surface area and diameter of the particles, was considered the dominant mechanism of the release of PPZ from nanoparticles [60].

### 3.6. Solid State Characterization

#### 3.6.1. Differential Scanning Calorimetry (DSC)

**Figure 4:**
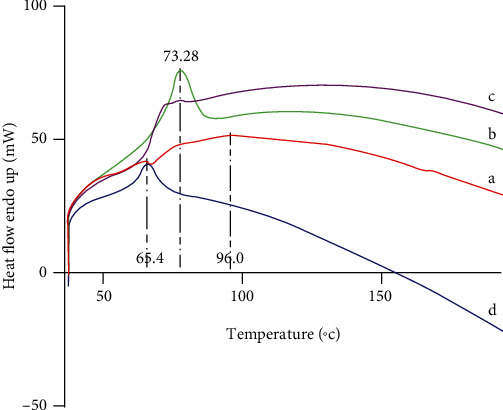
Thermograms of the (a) PPZ, (b) GMS, (c) physical mixture of PPZ and GMS, and (d) PPZ-SLN.

DSC is a tool to investigate the melting and recrystallization behavior of crystalline materials like SLNs. The DSC thermograms of the PPZ, lipid, physical mixture of the drug with lipid (1 : 1), and lyophilized PPZ-SLN are shown in Figure 4. The thermal curve of PPZ showed a broad endothermic effect that reached the maximum at 96°C, corresponding to the melting point of the drug. The pure lipid (GMS) showed a sharp endothermic peak at the melting point of 73.28°C. In the DSC profile of the physical mixture of the GMS and drug, a peak that resembled the superimposition of both peaks was apparent as a low-intensity endothermic peak related to the lipid and a broad endothermic effect related to the drug. In the DSC profile of lyophilized PPZ-SLN, the complete disappearance of the drug peak and a shift in the endothermic peak of lipid were observed, indicating an amorphous solid dispersion. It also could indicate that the drug is completely dispersed in the formulation and the amount of the drug is inadequate in the lyophilized product to show an endothermic peak [61]. A reduction in the enthalpy of the melting endotherm was noticed when the lipid was present in the composition of the PPZ-SLN. Besides, the lipid melting point shifted to 65.4°C, demonstrating a lower temperature compared to the bulk lipid. The melting point reduction might be connected to the embodiment of PPZ molecules in the SLN, resulting in increased imperfections of the crystal lattice of the lipid and reduction of the melting point [61–64].

#### 3.6.2. Powder X-Ray Diffractometry (P-XRD)

**Figure 5:**
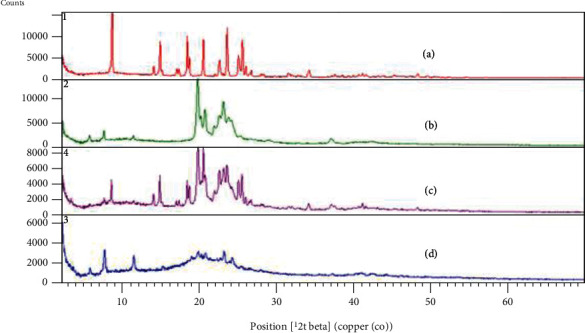
XRD spectra of the (a) PPZ, (b) GMS, (c) physical mixture of PPZ and GMS, and (d) PPZ-SLN.

P-XRD patterns of the pure drug (PPZ), lipid (GMS), physical mixture of the drug with lipid (1 : 1), and lyophilized PPZ-SLN are demonstrated in Figure 5. Powder-XRD patterns of PPZ showed sharp peaks at 2*θ* scattered angles of 8.540°, 13.93°, 14.75°, 18.29°, 18.55°, 20.38°, 22.47°, 23.44°, 23.59°, 24.91°, and 25.43°, confirming its crystalline nature. The absence of these diffraction peaks in lyophilized SLN points out the amorphous condition of the drug in the PPZ-SLN sample. The sharp peaks of lipid in the range of 19-25° are related to the high crystallinity of GMS. The reduced intensity of these peaks in the lyophilized SLN pattern indicates a decrease in the degree of the crystallinity of lipid during formulation and lyophilization of SLN. The lipid had formed a less ordered crystal lattice to develop a capacity for the drug to be embedded [61, 62, 65]. The crystallinity percentages of the PPZ, GMS, physical mixture, and SLN values are determined as 22.5, 26.3, 12.7, and 6.49, respectively. This reduction in crystallinity was confirmed with DSC analysis [61]. The P-XRD pattern of the physical mixture resembled the superimposition of the pure drug and the lipid peaks. The drug and lipid crystallinity peaks are detectable in the physical mixture, indicating no incompatibility between them [65].

#### 3.6.3. FTIR

**Figure 6:**
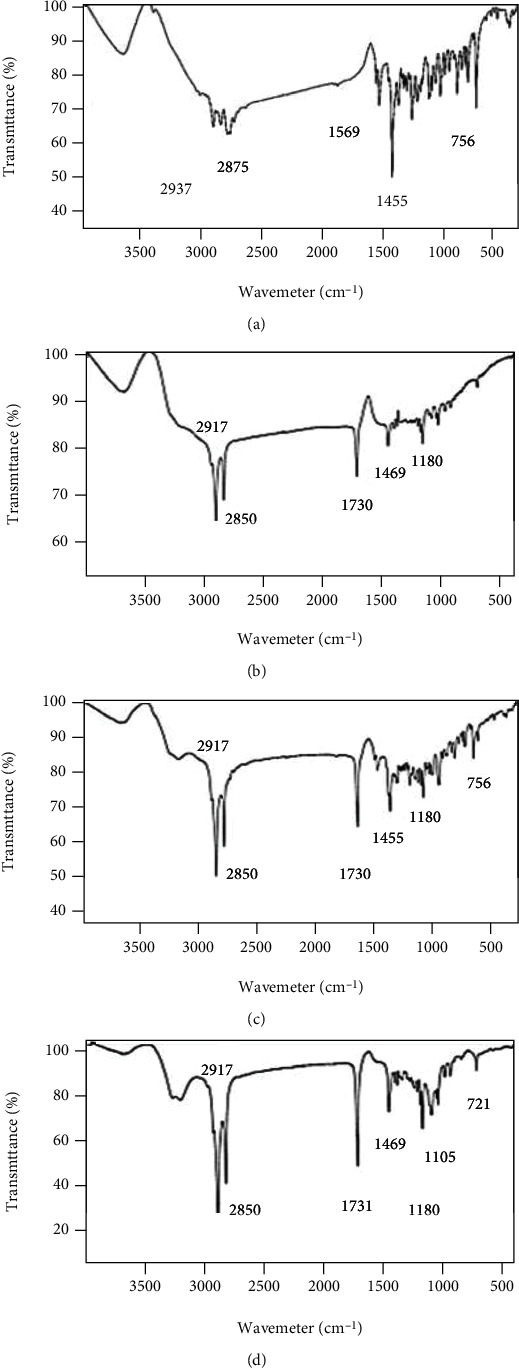
FTIR spectrum of the (a) PPZ, (b) GMS, (c) physical mixture of the drug and lipid, and (d) PPZ-SLN.

The FTIR technique allows the detection of new band formation in the solid phases of SLNs. The formation of new bands represents new substance formation and the incompatibility of excipients in SLN. The FTIR spectra of the PPZ, GMS, PPZ-SLN, and physical mixture of the drug and lipid are shown in Figure 6. Characteristic bands of the FTIR spectrum of PPZ are explained as follows. The sharp bands at the wavenumbers of 1455.6 cm^−1^ and 1569 cm^−1^ represent C=C aromatic stretching. C-C stretching bands are visible at 2875 cm^−1^ and 2937 cm^−1^, and C-Cl stretching is manifested as a sharp band at 756 cm^−1^ [66]. The presence of characteristic peaks of PPZ and the absence of new peaks in both the PPZ-SLN and the physical mixture of the drug and lipid indicated the compatibility between the drug and the lipid. Based on DSC and FTIR analysis, it can be concluded that there is no evidence for the formation of new bands between the drug and the lipid [65, 67, 68].

### 3.7. Cytotoxicity Studies

**Figure 7:**
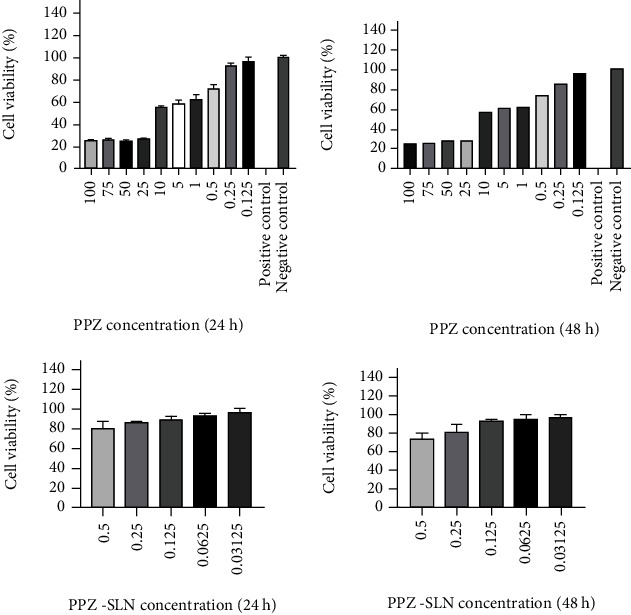
HT-29 cell viability assessed by MTT assay treated with free PPZ solution (24 h postincubation; 48 h post-incubation).

The viability of HT-29 cells was determined after 24- and 48-hour post-incubation of cells with optimized PPZ-loaded SLNs and free PPZ in the serum-free medium (Figure 7). As shown in the figure, both nanoparticles and free PPZ solution exhibited concentration-dependent cytotoxicity. Moreover, as it is obvious from the figures, the cell viabilities in the appropriate concentrations were not significantly different between nanoparticles and free PPZ (*p* > 0.05), indicating insignificant cytotoxicity of the nanocarrier system on the studied cell line. The positive control represents the treated cells with DMSO, and the negative control indicates the treated cells with the culture medium. IC_50_ values of PPZ solution for 24 and 48 h treatment in HT-29 cells, respectively, are 8.30 *μ*g/ml and 7.27 *μ*g/ml. Also, IC_50_ values of PPZ-containing nanoparticles in HT-29 cells for 24 and 28 h treatment are 7.06 *μ*g/ml and 7.09 *μ*g/ml, respectively. In conclusion, the lack of cytotoxicity of the optimized SLN formulation was observed on HT-29 cells using MTT assay. The obtained results suggested that the prepared SLNs were well tolerated and considered safe and highly biocompatible drug carriers.

## 4. Conclusions

In this study, SLNs intended for oral drug delivery of PPZ were successfully prepared by the solvent emulsification-evaporation method. It was shown that PPZ as a lipophilic drug could be favorably incorporated into the lipid core of the nanoparticles. The design of experiments was utilized to interpret the interactions of formulation variables on the physicochemical properties of nanoparticles. Using statistical optimization, the optimized nanoparticles exhibiting small diameters with a narrow size distribution, proper zeta potential, and a high percentage of drug encapsulation were obtained. An *in vitro* release study showed a sustained release of the drug from nanoparticles. Finally, the prepared SLNs can be considered a novel strategy for improving the bioavailability of PPZ by accessing the lymphatic pathway to avoid the first-pass metabolism.

## Figures and Tables

**Table 1:** Variables used in the Box-Behnken experimental design.

Independent variables (factors)		Levels	
		-1	+1
Numeric factors	Concentration of Tween 80 (*A*)	0.5 mg/ml	2.0 mg/ml
	Amounts of lecithin (*B*)	100 mg	300 mg
	Amounts of GMS (*C*)	100 mg	500 mg
Dependent variables (responses)			
*Y*_1_ = size (nm)			
*Y*_2_ = zeta potential (mV)			
*Y*_3_ = entrapment efficiency (EE%)			

**Table 2:** Box-Behnken experimental design runs. Results are given as mean ± SD (*n* = 3).

Independent variables				Dependent variables		
No.	Tween 80 conc. (*A*) (mg/ml)	Lecithin conc. (*B*) (mg)	GMS conc. (*C*) (mg)	Size (*Y*_1_) (nm)	ZP (*Y*_2_) (mV)	EE (*Y*_3_) (%)
1	1.25	100	100	147.90 ± 16.96	−21.2 ± 2.21	75.02 ± 0.99
2	0.50	200	500	96.15 ± 3.37	−26.46 ± 1.41	87.5 ± 1.65
3	0.50	300	300	116.66 ± 2.91	−32.13 ± 3.43	71.39 ± 5.53
4	0.50	200	100	142.43 ± 3.49	−33.16 ± 3.23	72.53 ± 3.66
5	1.25	200	300	146.46 ± 6.44	−27.56 ± 2.87	51.40 ± 6.70
6	2.00	200	100	115.76 ± 3.02	−32.26 ± 3.56	36.17 ± 3.76
7	1.25	300	500	215.10 ± 13.38	−28.30 ± 3.37	47.16 ± 5.80
8	1.25	100	500	131.60 ± 10.87	−24.16 ± 0.81	84.86 ± 1.23
9	0.50	100	300	223.03 ± 12.98	−23.96 ± 0.85	84.73 ± 3.04
10	1.25	200	300	197.23 ± 14.57	−26.70 ± 1.38	77.11 ± 2.43
11	2.00	300	300	181.53 ± 10.95	−31.36 ± 3.76	71.56 ± 1.25
12	2.00	100	300	164.33 ± 27.81	−24.03 ± 3.43	78.4 ± 1.73
13	2.00	200	500	264.36 ± 12.37	−26.13 ± 0.20	76.13 ± 1.44
14	1.25	300	100	188.43 ± 12.71	−35.8 ± 1.32	71.53 ± 3.74
15	1.25	200	300	202.30 ± 8.14	−26.7 ± 0.60	86.10 ± 10

**Table 3:** Characteristics of the models fitted to responses.

Dependent variables (responses)	Best-fitted model	*R*-squared	Adj *R*-squared	Pred *R*-squared	Adeq *R*-squared
Particle size (*Y*_1_)	2FI model	0.6461	0.5496	0.3916	10.099
ZP (*Y*_2_)	Linear model	0.7785	0.7416	0.5791	13.804
EE% (*Y*_3_)	Quadratic model	0.5262	0.4472	0.3244	5.093

**(a):** 

Optimized independent variables				Predicted dependent variables (responses)		
No.	Tween 80 conc. (*A*) (mg/ml)	Lecithin conc. (*B*) (mg)	GMS conc. (*C*) (mg)	*Y*_1_ = size (nm)	*Y*_2_ = zeta potential (mV)	*Y*_3_ = EE%
1	0.5	196.23	500.00	96.39	-25.66	84.13

**(b):** 

Observed responses					
Size (nm)		ZP (mV)		EE%	
Observed response (mean ± SD)	Prediction error (%)	Observed response (mean ± SD)	Prediction error (%)	Observed response (mean ± SD)	Prediction error (%)
105.56 ± 3.92	8.69	−28.64 ± 2.28	10.32	83.28 ± 1.29	-1.02

**Table 5:** Mathematical modeling of PPZ released from SLNs.

	Zero-order	First-order	Second-order	Diffusion	Hixson-Crowell	Baker-Lonsdale	Chosen “*r*”
*A*	-3.6100821	0.75263427	-0.03793993	-30.9569	-0.230039	-0.03765568	0.965741338
*B*	2.60180233	0.00639024	0.007660334	19.8139	0.08247552	0.009795867	
*R*	0.938569	0.105767	0.9454375	0.9407	0.965741	0.9608071	
*K*	2.60180233	0.01471671	0.007660334	19.8139	0.08247552	0.009795867	
*t* (1/2)	19.2174476	47.0893131	1.30542605	6.36797	11.5933463	5.614612727	

## Data Availability

Data are available on request.
